# Noninvasive Diagnosis Revealed a Strong Therapeutic Response and Skin Barrier Changes in Hemoporfin‐Mediated Photodynamic Therapy for Port‐Wine Stains

**DOI:** 10.1111/srt.70369

**Published:** 2026-06-15

**Authors:** Kai Chen, Jin‐Zhao Wu, Yan‐Yan Hu, Qian Jiang, Yu‐Xin Xia, Jie Chen, Lan Chen, Liu‐Qing Chen, Dong‐Sheng Li

**Affiliations:** ^1^ Department of Dermatology, Traditional Chinese and Western Medicine Hospital of Wuhan, Tongji Medical College Huazhong University of Science and Technology Wuhan China; ^2^ Hubei Key Laboratory of Infectious and Immune Skin Diseases, Wuhan No. 1 Hospital, Tongji Medical College Huazhong University of Science and Technology Wuhan China; ^3^ Department of Dermatology, The Sixth Hospital of Wuhan Affiliated Hospital of Jianghan University Wuhan China

**Keywords:** optical coherence tomography (OCT), photodynamic therapy (PDT), port‐wine stains (PWS), reflectance confocal microscopy (RCM), therapeutic evaluation

## Abstract

**Background:**

Noninvasive skin imaging, such as reflectance confocal microscopy (RCM) and optical coherence tomography (OCT), has been extensively used to collect objective clinical data and assess the morphological and ultrastructural change of tissue. However, the application of these noninvasive diagnosis techniques for the therapeutic evaluation of hemoporfin‐mediated photodynamic therapy (HMME‐PDT) in port‐wine stains (PWS) has rarely been reported.

**Objectives:**

To explore the clinical efficacy of HMME‐PDT for PWS, and assess the application of RCM and OCT in objective assessment of the efficacy of HMME‐PDT.

**Methods:**

Clinical images, RCM findings, and OCT scans were used to evaluate the treatment efficacy. The vascular diameter and density under RCM findings, the vascular density, and depth of OCT scans were used for objective therapeutic evaluation. Skin surface roughness and barrier functions were used to analyze the skin barrier changes.

**Results:**

After PDT treatment, the clinical pictures showed excellent improvements, and the RCM findings revealed significantly decreased vascular diameter (100.0 ±2.450 vs. 59.61± 4.021, *p*<0.05) and density (16.79 ± 0.6568 vs. 10.91 ± 0.8052) in treated areas. Moreover, the OCT scans further confirmed the decrease in vascular density and depth, and the skin surface roughness of Ra (arithmetic mean), Rz (the average depth of roughness), Rq (the mean square roughness) and the skin barrier function of transepidermal water loss (TEWL), stratum corneum hydration (SCH), and potential of hydrogen (pH) of these treated areas all were significantly changed (*p* < 0.05).

**Conclusions:**

HMME‐PDT is an effective and safe treatment option for PWS patients, and noninvasive diagnosis combined with clinical images are excellent techniques for objectively evaluating efficacy of HMME‐PDT.

## 1 Introduction

Port‐wine stains (PWS) is a common vascular malformation that often occurs on the face and neck, with an incidence of 3–5/1000 in neonates globally [1]. The skin lesion usually cannot resolve spontaneously and the progression of lesion even can lead to a variety of syndromes, which seriously affect the physical and mental health of patients [2]. Hemoporfin‐mediated photodynamic therapy (HMME‐PDT) is a non‐invasive, vascular‐targeted therapy for the treatment of PWS, which is now widely used in China due to its safe and good therapeutic effects [3, 4, 5]. At present, histopathology examination and dermatologist evaluation are the main methods to assess the clinical efficacy of treatment for PWS, but the histopathology is invasive and the dermatologist evaluation is subjective and small improvements are difficult to detect with naked eyes [6]. Therefore, some objective and non‐invasive methods are required. Previous studies have reported that certain skin characteristics, such as average epidermal thickness or depth of PWS lesions, are associated with treatment outcomes [7]. Although numerous non‐invasive diagnostic techniques such as dermoscopy, laser speckle contrast imaging (LSCI), high‐frequency ultrasound (HFUS), and VISIA‐CR system have been applied to evaluate the clinical efficacy of the treatment for PWS in recent years [8, 9, 10], there is still lacking a detection technique for direct observation and quantitatively monitoring of skin structures at a more microscopic level.

Reflectance confocal microscopy (RCM) is a powerful non‐invasive imaging technique that uses an 830 nm semiconductor laser to provide in vivo tissue images at nearly cellular histological resolution [11, 12]. As reported, RCM is suitable for examining the morphology and structure changes of tissues, especially for observing the vascular diameter and depth of skin lesions in microns [13]. Thus, it is feasible to use RCM to assess the changes in the malformed blood vessels before and after PDT treatment, but the clinical application in dermatology is insufficient. Optical coherence tomography (OCT) is another non‐invasive optical imaging technique and is capable of producing micron‐sized, high‐resolution, and three‐dimensional images of biological tissues [14]. In recent years, the application of this technique has developed rapidly and has been extensively used in many fields, such as ophthalmology, cardiology, neurology, and cosmetic dermatology [15, 16, 17, 18]. Additionally, OCT also allows for the measurements of epidermal thickness and the quantitative analyses of vascular density and depth of skin [19]. Taken together, RCM and OCT are theoretically well‐suited for examination of skin vascular changes, but the application of these noninvasive diagnosis techniques to assess the treatment efficacy of HMME‐PDT in patients with PWS has rarely been reported.

In this study, we observed the clinical efficacy of HMME‐PDT for PWS patients, analyzed the changes in ectatic deformed vessel diameter, vascular density and depth of lesional skin using RCM and OCT, and examined the changes of skin barrier after PDT treatment, to assess the application of RCM and OCT in objective assessment of the treatment efficacy of PDT for PWS and to provide safety guidance for post‐treatment management.

## 2 Methods

### 2.1 Study Design

We recruited 33 patients with clinical diagnosis of PWS to receive HMME‐PDT treatment at the Dermatology Department of Wuhan No.1 Hospital (March 2022 to July 2023). Patients were excluded if have any one or more of the following conditions: (1) women who were currently pregnant or lactating; (2) other vascular malformations, vessel‐related syndromes, or other conditions that might interfere with the study; (3) allergy to porphyrins and analogues or history of medications that might cause photosensitivity disorders; and (4) history of treatment with laser or systemic treatment for PWS during the past 4 weeks.

In this study, PWS patients received different pain relief strategies during PDT, including cold‐air analgesia, oral anesthetics, and systemically administered analgesia. Moreover, PWS patients can also receive PDT treatment under general anesthesia condition. Before the treatment, HMME was intravenously injected steadily at a dose of 5 mg/kg for 20 min. Ten minutes after the onset of injection, the patient was irradiated with a 532 nm green LED light (LED Therapeutic Machine, LED‐IE, Wuhan YaGe Optic and Electronic Technique Co. Ltd., Wuhan, Hubei, China) at 85–95 mW/cm^2^ for 20–22 min (96–115 J/cm^2^). After the treatment, the skin lesions were cold sprayed, and the patient was instructed to avoid light exposure in the treated area for 2 weeks. Patients received different PDT sessions in this study: 22 patients received one PDT session, 6 patients received two PDT sessions, 4 patients received three PDT sessions, and 1 patient received four PDT sessions. Also, the interval between PDT sessions is 2 months in this study. All patients signed informed consent of study participation before the treatment, and this study was approved by the ethics committee of Wuhan No. 1 Hospital (Wuhan, China).

### 2.2 Efficacy Criteria

Before and after PDT treatment (8 weeks), the skin lesions were taken using the supplied digital camera (Canon SLR 650D, Tokyo, Japan) from three fixed angles (90° and 45° to the left and right of the treated surface) under the same light. The degree of improvement in the treated area was evaluated according to the following four levels: (i) Nearly completely resolved (CR): ≥ 90%, (ii) great improvement (GI): 60%–89%, (iii) some improvement (SI): 20%–59%, and (iv) no improvement (NI): < 20% [20, 21]. The treatment efficacy was evaluated and calculated (sum of the CR, GI, and SI) by three dermatologists in a blinded and independent manner. Patients with a variable score level among the three raters will be excluded in this study.

### 2.3 Therapeutic Evaluation

RCM images were taken using the VivaScope skin laser confocal scanning microscope (Luricl, USA). The vascular diameter and density of the RCM images were analyzed using the provided software. OCT images were scanned using the Vivo Sight OCT scanner (Michelson Diagnostics, UK). After scan, the OCT images were analyzed using the advanced VivoSight analysis software and the skin surface roughness was calculated using the three values as described previously [22].

### 2.4 Skin Barrier Function Determination

The skin barrier function indexes of transepidermal water loss (TEWL), stratum corneum hydration (SCH), and epidermal pH before and after PDT treatment were measured in a room with constant temperature (22–24°C) and humidity (45%–65%). TEWL was measured by a skin moisture loss tester (Tewameter TM300, Courage & Khazaka, Cologne, Germany), SCH and pH were measured by a skin moisture tester (Derma Unit SSC3, Courage & Khazaka, Cologne, Germany). Each area was measured three times to calculate the average value.

### 2.5 Statistical Analysis

GraphPad Prism 9 (GraphPad Software, San Diego, CA, USA) was used for all statistical analyses. Paired *t* test was applied to evaluate the changes in vascular diameter and density, depth of blood vessel, Ra, Rz, Rq, TEWL, SCH, and epidermal pH of PWS lesions between groups before and after PDT treatment. The results were expressed as mean value ± standard error of the mean (mean ± SEM) and *p* value < 0.05 was considered statistically significant.

## 3 Results

### 3.1 Good Treatment Efficacy of HMME‐PDT in PWS Patients

**FIGURE 1:**
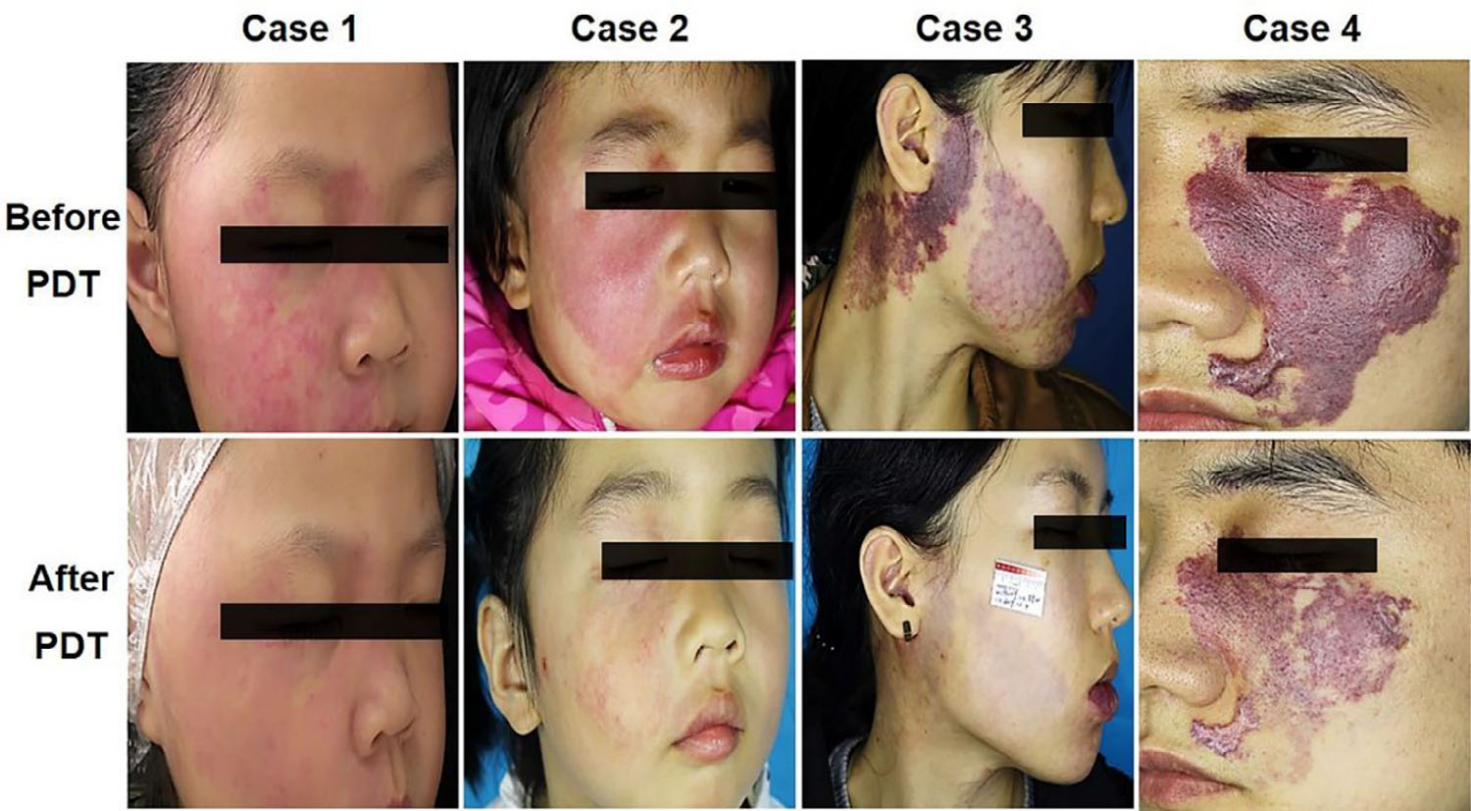
Representative clinical images showed a good therapeutic response of hemoporfin‐mediated photodynamic therapy in port‐wine stains. Upper panel: Representative images of different types of port‐wine stains (PWS) in the patients before photodynamic therapy (PDT). Lower panel: After PDT treatment, the clinical pictures showed excellent improvements of the treated areas. Eight weeks after PDT treatment, the photographs were taken. PDT sessions: Case 1 (1), Case 2 (2), Case 3 (3), and Case 4 (4).

A total of 33 patients (14 females) with a mean age of 23.03 ± 11.46 years were included in this study. There were 3 (9.09%) cases with pink, 19 (57.58%) cases with red, 9 (27.27%) cases with purple, and 2 (6.06%) cases with hypertrophic‐type PWS. For all patients, the average treatment time was 1.515 ± 0.972. More importantly, after the treatment sessions, 3 (9.09%) patients were found to be nearly CR, 13 (39.39%) patients were GI, 16 (48.49%) patients were SI, and only 1(3.03%) patient was NI. These results, which are summarized in Table 1, indicated a good treatment efficacy (96.97%) of HMME‐PDT in PWS patients. Moreover, some representative clinical images of PWS patients before and after PDT treatment are presented in Figure 1.

**TABLE 1:** Demographic characteristics of the 33 PWS patients in this study.

Characteristic	Value	Percentage (%)
Gender		
Male	19	57.58
Female	14	42.42
Age (years)	23.03±11.46	
Localization		
Head and neck	28	84.85
Arm or leg	4	12.12
Trunk	1	3.03
Color of PWS		
Pink	3	9.09
Red	19	57.58
Purple	9	27.27
Hypertrophic	2	6.06
Treatment times	1.515±0.972	
Treatment response		
NI	1	3.03
SI	16	48.49
GI	13	39.39
CR	3	9.09

*Note*: Treatment response: no improvement (NI): < 20%; some improvement (SI): 20%–59%; great improvement (GI): 60%–89%; nearly completely resolved (CR): ≥ 90%.

### 3.2 Reflectance Confocal Microscopy (RCM) Findings Revealed the Decreased Vascular Diameter and Density After PDT Treatment

**FIGURE 2:**
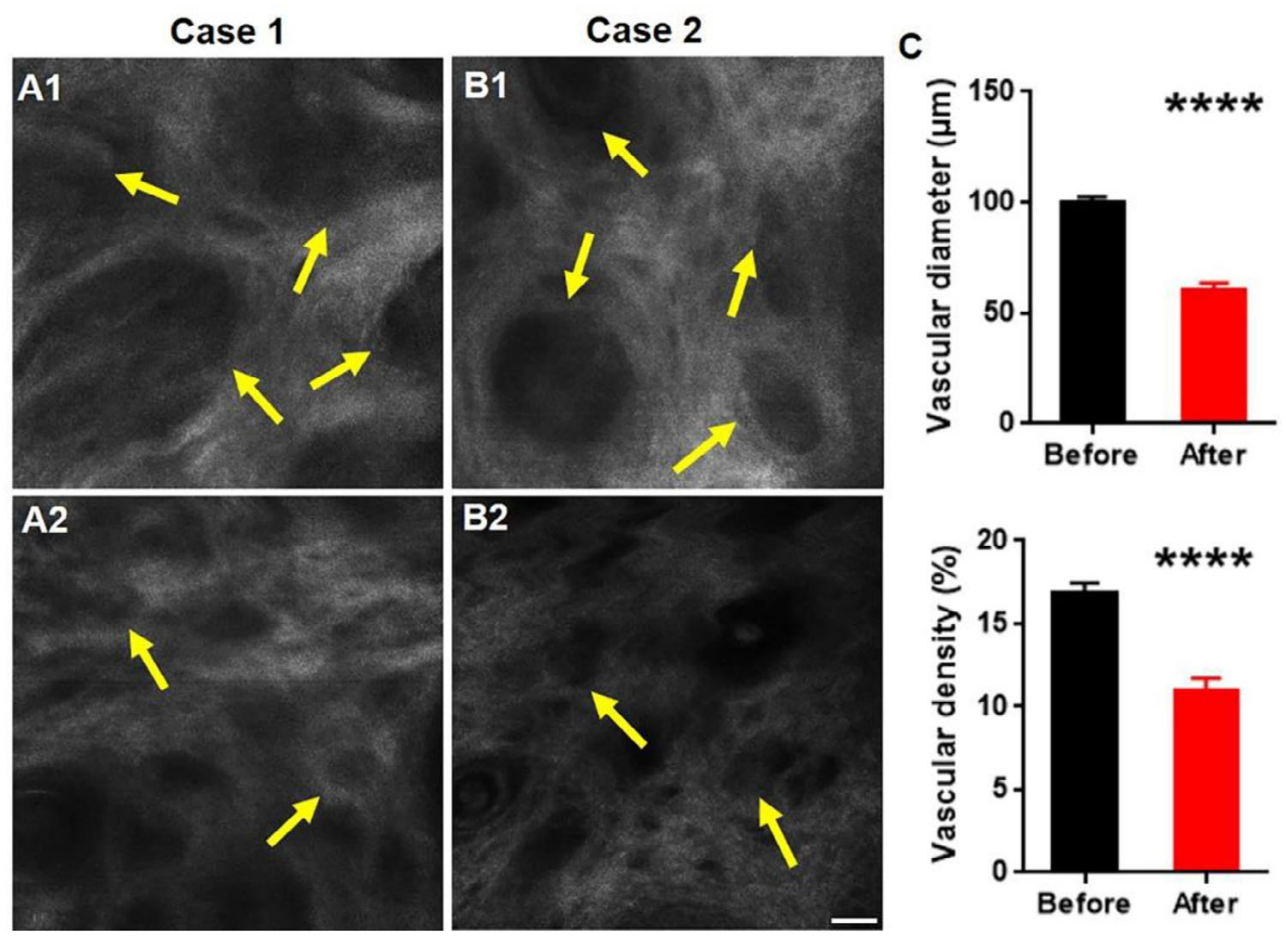
Reflectance confocal microscopy (RCM) findings revealed the decreased vascular diameter and density after photodynamic therapy (PDT) treatment. (A1, B1) Before PDT, the RCM images obviously showed the expansive blood vessels in the dermis of two patients. (A2, B2) After PDT treatment, the number of dilated vessels and the vascular diameter both were markedly reduced. Yellow arrows indicated the malformed vessels in dermis. Scale bar = 50 µm. PDT sessions: Case 1 (2) and Case 2 (3). (C) Quantitative analysis of the vascular diameter and density of skin lesions before and after PDT treatment. *n* = 33 for each group, results are shown as mean ± SEM, paired sample *t* test, **** *p* < 0.0001.

To determine the morphological as well as the clinical symptoms changes, we performed RCM scan of treated areas before and after PDT treatment. Under microscopy, we can see the expansive blood vessels in the dermis of PWS patients. After PDT treatment, the number of dilated vessels and the vascular diameter both were reduced (Figure 2A, B), which revealing the remarkable efficacy of PDT in PWS patients. Moreover. statistical analysis also showed a significant decrease in the vascular diameter (100.0 ±2.450 vs. 59.61± 4.021, *p*<0.05) (95% CI: −0.8270 to −0.5945, *p*< 0.0001) and density (16.79 ± 0.6568 vs. 10.91 ±0.8052, *p*<0.05) (95% CI: −0.7190 to −0.3898, *p* < 0.0001) of treated areas after PDT treatment (Figure 2C). Collectively, the decreased vascular diameter and density objectively reflected the therapeutic efficacy of PDT in PWS patients.

### 3.3 Optical Coherence Tomography (OCT) Scans Further Confirmed the Decreased Vascular Density and Depth After PDT Treatment

**FIGURE 3:**
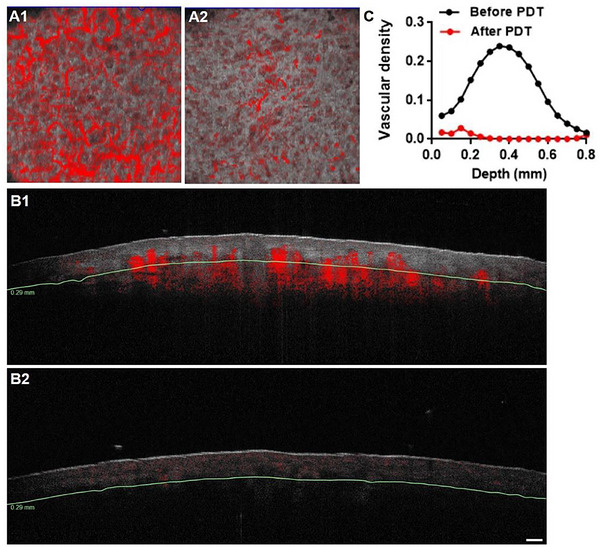
Optical coherence tomography (OCT) scans further confirmed the decreased vascular density and depth after photodynamic therapy (PDT) treatment. Typical OCT image of a patient (PDT session: 1) with dilated vessels before (A1, B1) and after (A2, B2) PDT treatment. Scale bar = 200 µm. (C) OCT imaging data showed that the vascular density at the corresponding depth was obviously decreased after PDT treatment as compared with those before PDT.

**FIGURE 4:**
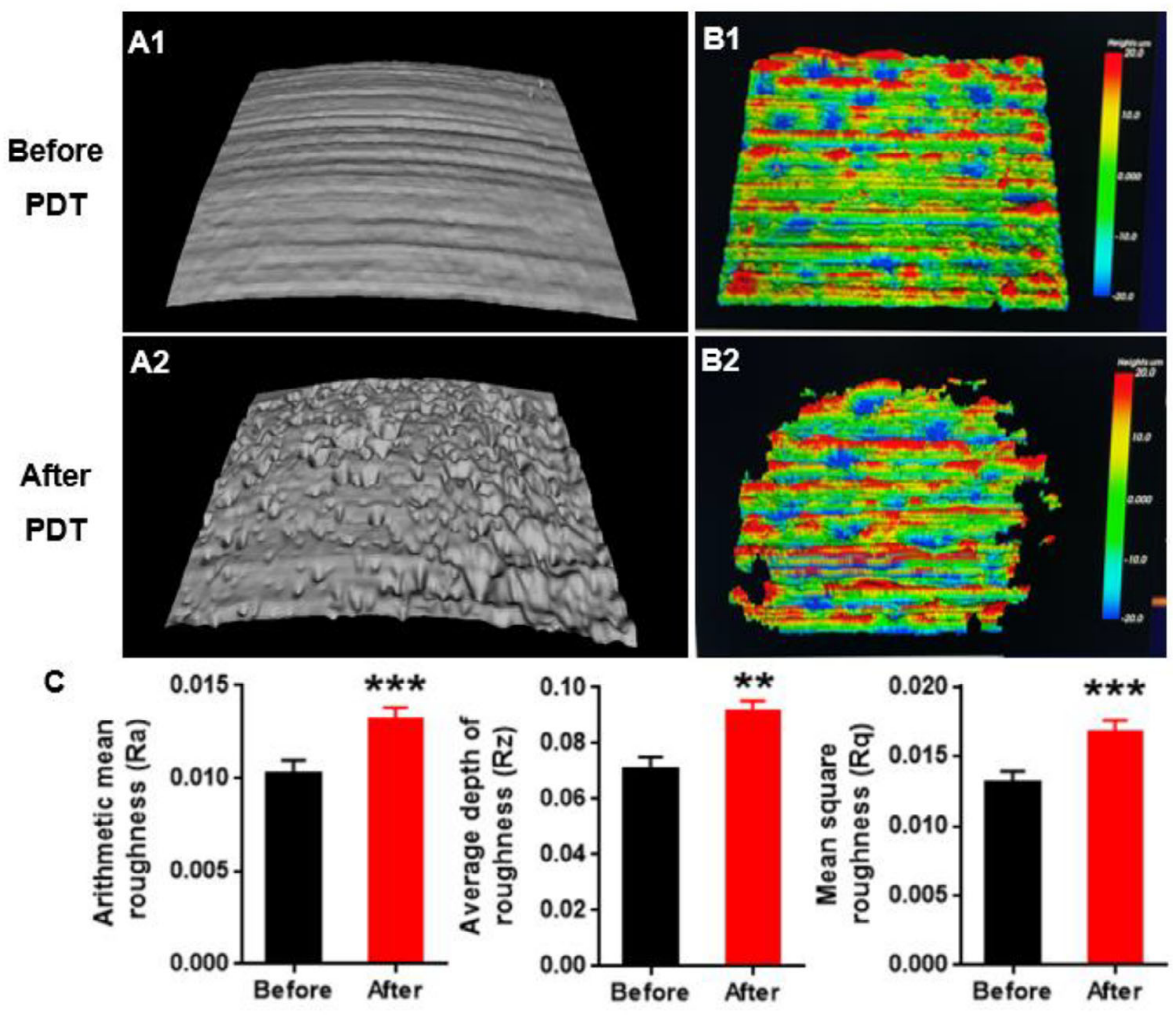
Photodynamic therapy (PDT) caused obviously skin surface roughness changes in treated areas. Representative three‐dimensional (3D) optical coherence tomography (OCT) of surface roughness in a patient (PDT session: 1) before (A1) and after (A2) PDT treatment, B1 and B2 represent the corresponding heat map. (C) Comparison of the changes in Ra, Rz, and Rq at the treated area before and after PDT treatment. *n* = 33 for each group, results are shown as mean ± SEM, paired *t* test, ** *p* < 0.01, *** *p* < 0.001.

To further confirmed the ultrastructural alterations, we then performed OCT scan of these treated areas. OCT imaging data of a typical patient showed that the vascular density at the corresponding depth is significantly reduced after PDT treatment (Figure 3A). Moreover, the depth of blood vessels of treated areas also was obviously decreased (Figure 3B, C). These results further confirmed the decreased vascular density and depth of treated areas, and a good therapeutic response of PDT in PWS patients. After PDT treatment, we also noted that the OCT image showed obviously villus‐like protrusions on the surface of skin (Figure 4A), and the corresponding heat map confirmed the damage of skin surface (Figure 4B). Meanwhile, the surface roughness of Ra (0.01026 ± 0.0006955 vs. 0.01313 ± 0.0006815, *p*<0.05) (95% CI: 0.1135–0.5426, *p* < 0.01), Rz (0.07080 ± 0.004255 vs. 0.09110 ± 0.004035, *p*<0.05) (95% CI: 0.1715–0.5831, *p* < 0.001) and Rq (0.01306 ± 0.0008563 vs. 0.01673 ± 0.0008361, *p*<0.05) (95% CI: 0.1268–0.5521, *p* < 0.01), and the skin barrier function of TEWL, SCH, and pH of treated areas all were significantly changed (Figure 4C, Table 2). Thus, the OCT imaging data further confirmed the good therapeutic effect of PDT and revealed the skin barrier changes in treated areas.

**TABLE 2:** Comparison of skin barrier function before and after PDT treatment in PWS patients (mean ± SEM).

Characteristic (*n* = 33)	Before	After	*p* value
TEWL (g/(h·m^2^))	9.823 ± 0.9325	14.65 ± 1.897	0.0064
SCH	69.45 ± 3.412	58.77 ± 4.437	0.0153
pH	5.670 ± 0.06001	5.833 ± 0.04953	0.0002

Abbreviations: pH, potential of hydrogen; SCH, stratum corneum hydration; TEWL, transepidermal water loss.

All patients developed various kinds of treatment reactions including edema, purpura, blister, and scab, and no serious systemic adverse reactions were observed. Taken together, our clinical data demonstrated that PDT is an effective and safe treatment option for PWS patients, and noninvasive diagnosis (RCM and OCT) combined with clinical images are excellent techniques for objectively evaluating efficacy of PDT.

## 4 Discussion

We retrospective analyzed the clinical efficacy of 33 PWS patients who received HMME‐PDT treatment in this study. After the treatment sessions, 32 (96.97%) patients were found to be effective improvement (≥20%) and only 1 (3.03%) patient was NI, and none of patients were observed obvious systemic adverse reactions. In line with our data, previous studies also reported the high efficacy and safety data of HMME‐PDT for PWS patients [23, 24]. Previous studies also reported that the number of PDT sessions is associated with the lesion subtype, and the purple‐type PWS responds worse to the treatment than the pink‐type PWS [4, 25], so the patients received different PDT sessions in this study. Furthermore, previous studies reported that vascular changes in mean diameter and density under RCM accurately reflected the treatment efficacy of PWS lesions [9, 26]. As expected, our results shown that both the vascular diameter and density under RCM were obviously decreased after treatment as compared with those before treatment. Thus, these results further confirming the good therapeutic effect of PDT in patients with PWS. Therefore, our clinical data support the conclusion that HMME‐PDT is an effective and safe treatment therapy for PWS patients.

At the same time, the RCM pattern of vascular diameter and density of skin lesions was associated with the clinical outcomes to previous treatment [27, 28]. After PDT treatment, we also observed that the average vascular diameter decreases from 100.0 ± 2.450 µm to 59.61± 4.021 µm, and the average vascular density decreases from 16.79 ± 0.6568 to 10.91 ± 0.8052, respectively. These objective changes accurately reveal the good improvements and outcomes of PDT for PWS. Moreover, quantitatively monitoring the OCT scans before and after PDT treatment also showed a significant reduction in vascular density at the corresponding depth and a conspicuous decrease in vascular depth of treated areas. These results suggest that the superficial blood vessels in PWS patients have been successfully targeted for destruction, and providing further evidences of the remarkable efficacy of HMME‐PDT in treating PWS patients. Similarly, recent clinical studies utilized OCT to evaluate the treatment efficacy of HMME‐PDT in patients with PWS by objectively analyzing the changes in vascular diameter and depth [19, 29]. Thus, both RCM and OCT are good non‐invasive methods to assess the clinical response of PWS patients by providing visualizable and reproducible in vivo images and accurate clinical data.

In addition to identifying blood vessels and morphological changes, high‐resolution OCT imaging also offered quantify changes in skin surface roughness [30, 31]. In this study, we found that the skin roughness of Ra, Rz, and Rq all are significantly increased after PDT treatment, indicating the change of skin surface in treated areas. Noticeably, previous observations suggested that the ultrastructural changes in skin roughness might contribute to the skin barrier damage [32]. We then examined the epidermal barrier function of TEWL, SCH, and pH of PWS patients. Compared to pretreatment, our results showed a significant increase in TEWL and pH, and a remarkable decrease in SCH in treated areas. Collectively, changes in skin roughness and barrier functions further confirmed the damage of skin barrier in PWS patients after PDT treatment. More importantly, previous study reported that patients with impaired skin barriers are prone to be dry skin and increasing the prevalence and risk of dermatitis and skin infection [33]. Therefore, we recommended that PWS patients should use some skin protection creams to repair the skin barrier after PDT treatment.

In conclusion, our study demonstrated that HMME‐PDT is an effective and safe treatment therapy for PWS patients, and clinical images combined with morphological changes of malformed vessels of skin lesions under RCM and OCT scans before and after HMME‐PDT, providing an objective and accurate assessment of the treatment efficacy. Meanwhile, our results also reported the impaired skin barrier of treated areas and showed that it is necessary for PWS patients to repair the skin barrier after PDT treatment. Because of the relatively small sample size and the single center in this study, we will perform multi‐center observation to provide a better therapeutic evaluation system for HMME‐PDT treatment of PWS patients in the future.

## Ethics Statement

This study was reviewed and approved by the Ethics Committee of Wuhan No. 1 Hospital, Tongji Medical College, Huazhong University of Science and Technology.

## Consent

All patients signed informed consent of study participation before the treatment.

## Conflicts of Interest

The authors declare no conflicts of interest.

## Data Availability

The data that support the findings of this study are available on request from the corresponding author. The data are not publicly available due to privacy or ethical restrictions.
